# Mapping the flow of knowledge as guidance for ethics implementation in medical AI: A qualitative study

**DOI:** 10.1371/journal.pone.0288448

**Published:** 2023-11-02

**Authors:** Magali Goirand, Elizabeth Austin, Robyn Clay-Williams

**Affiliations:** Australian Institute of Health Innovation, Macquarie University, Sydney, Australia; Instituto Mexicano del Seguro Social, MEXICO

## Abstract

In response to the COVID-19 crisis, Artificial Intelligence (AI) has been applied to a range of applications in healthcare and public health such as case identification or monitoring of the population. The urgency of the situation should not be to the detriment of considering the ethical implications of such apps. Implementing ethics in medical AI is a complex issue calling for a systems thinking approach engaging diverse representatives of the stakeholders in a consultative process. The participatory engagement aims to gather the different perspectives of the stakeholders about the app in a transparent and inclusive way. In this study, we engaged a group of clinicians, patients, and AI developers in conversations about a fictitious app which was an aggregate of actual COVID-19 apps. The app featured a COVID-19 symptoms monitoring function for both the patient and the clinician, as well as infection clusters tracking for health agencies. Anchored in Soft Systems Methodology and Critical Systems Thinking, participants were asked to map the flow of knowledge between the clinician, the patient, and the AI app system and answer questions about the ethical boundaries of the system. Because data and information are the resource and the product of the AI app, understanding the nature of the information and knowledge exchanged between the different agents of the system can reveal ethical issues. In this study, not only the output of the participatory process was analysed, but the process of the stakeholders’ engagement itself was studied as well. To establish a strong foundation for the implementation of ethics in the AI app, the conversations among stakeholders need to be inclusive, respectful and allow for free and candid dialogues ensuring that the process is transparent for which a systemic intervention is well suited.

## Introduction

The COVID-19 crisis accelerated the usage of Artificial Intelligence (AI) in healthcare, fostering early development of innovative applications such as rapid case identification or digital epidemiological surveillance [1]. Even before COVID-19, there were concerns about medical AI perpetuating entrenched systemic inequalities. However, the rapid introduction of new applications during COVID-19 exacerbated these risks [2]. Given the rapid uptake of AI in healthcare apps, it is more critical than ever that we explicitly address how ethics principles are applied despite the urgency of the COVID-19 crisis [3,4].

There have been several iterations of ethics principles and guidelines for AI developed by technology providers (e.g., IBM), consulting firms (e.g., PriceWaterCooperhouse), professional associations (e.g., Institute of Electrical and Electronics Engineering) and governmental initiatives (e.g., The Villani Mission) [5]. These iterations move toward an “ethics by design” approach, advocating for implementing ethics guidelines at the conception of an AI application [4], and with the latest guidelines, such as those issued by the EU, involving stakeholders in the early phase of design [6]. While there have been reports of such approaches in the literature earlier on [7–10], guidance on resolving conflicts or tensions between ethics principles is limited [11].

Implementing ethics guidelines in AI-based Healthcare Applications (AIHA) is a complex issue, that is a situation where parameters are not all known making it difficult to link cause and effect, and for which there is no one size fits all approach [11]. Recent research demonstrates that the quality of the consultative process is important to establish a strong foundation to the implementation of the ethics guidelines [12]. A systems approach, also called systemic intervention, weaving different Systems Thinking methodologies, is well suited to address complex situations using a participatory process [13]. The approach in the context of this study involves using a model to support the conversations among stakeholders, anchored in Soft Systems Methodology (SSM) [14], and a set of questions to understand the boundaries of the system being explored, based on Critical Systems Heuristics (CSH) [15]. SSM provides a method to explore different worldviews using models representing the situation. The model, while incomplete because the complexity of the situation prevents us from knowing everything about it, supports the discussion among stakeholders. CSH is a heuristic that explores sources of motivation, power, knowledge, and legitimacy, so is particularly adapted to question ethical issues arising within a complex situation. In addition, Goirand et al found that the flow of knowledge between agents of the AIHA, that is not only the information conveyed between the agents but also *how* it is conveyed, could illuminate ethical issues [12]. Goirand et al developed a model and questions to support the conversations have then been developed to map the flow of knowledge within the system under consideration.

The COVID-19 crisis and the amplification of entrenched systemic inequalities has made the question of how to implement ethics in medical AI even more pressing and acute. The aim of this study, therefore, is to explore a process for implementing ethics in an AIHA based on techniques and principles, using a systems approach based on SSM and CSH. In this study, we set up a consultative process with a range of stakeholders looking at the flow of knowledge between the different agents of an AIHA. The exploration sought to answer questions about the process itself and the outcomes of the process: (1) How empowered do the participants feel? (2) How sustainable is the process? (3) How is collaboration enacted? (4) What improvements to the ethics framework upon which the app design is based are brought forth by the collaborative process? (5) How is ethics instilled in the design of the app? (6) Who are the custodians of the process?

## Methods

We invited a diverse group of people, representative of stakeholders, to explore the process for implementing ethics in an AIHA app. In addition, we set out to capture observations about the process itself and explore participants’ experience through exit interviews. This project was approved by the Faculty of Medicine, Health, and Human Sciences Low-risk Human Research Ethics Committee (HREC) at Macquarie University (project reference no. 520211098735884). The research team all identify as female with diverse backgrounds including engineering, psychology, and business. Two team members (EA & RCW) are experts in human factors, healthcare quality and safety. One team member (MG) was a PhD candidate having affinities with Eastern philosophy and Buddhism in particular. The three of them were systems thinking practitioners.

### Study design

Systemic intervention as defined by Gerald Midgley [13] which includes Soft Systems Methodology (SSM) and Critical Systems Heuristics (CSH), informed the design of this study which also built on a previous study carried out by the research team (see S1 File). SSM is well suited for tackling complex issues and advocates the usage of models representing the issue to support discussions [14]. The model is acknowledged as being an incomplete representation of reality and is used to surface worldviews and assumptions. Another tenet of SSM is that every stakeholder involved in the complex issue holds equally valuable knowledge. Because each stakeholder knows something, but none of them know everything, engaging them in a participatory process is a way to leverage the knowledge of each individual to trigger insights in the group. CSH is a philosophical framework to enquire about the ethical assumptions forming the boundaries of the situation under consideration such as the sources of power, expertise, motivation, and legitimacy [16]. It consists of a set of questions such as who is or should be the beneficiary of the app, who are or should be the decision makers, and who is or should be able to opt out of the app. The complete list of questions is available in Appendix B–CSH seed questions for group discussions in S1 Appendix. In the context of this study, the AIHA was represented as a system in a simple model. The conversations were oriented towards the flow of knowledge coursing through the system and the CSH boundary critique questions. To analyse the data, we used a reflexive methodology [16]. Reflexive methodology advocates the usage of different perspectives to look at the data, and the keeping of a reflective journal for the researchers. Such an approach aligns with the Systems Thinking design of the current study which strives to be inclusive of diverse points of view and considers the research as part of the process.

#### Development of the app

The app used in this study is a fictitious AIHA dedicated to helping patients sick with COVID-19 being treated remotely by their GP. A fictitious app was used for practical and legal reasons, and as such remains conceptual. The fictitious app is an aggregation of functionalities offered by real COVID-19 apps [17–19]. The app allows the patient to monitor some vital signs and communicate them to the GP along with answers to an in-app survey. The collected data is fed to a Machine Learning (ML) system. The data is used to advance diagnosis and treatment of the disease, better understand factors associated with infection, and recovery, and track clusters of infection. The system provides advice to General Practitioners (GP), and to health agencies. The goal is to help the GP make more informed decisions and follow their infected patients while avoiding physical contact.

#### Participant selection

**Fig 1:**
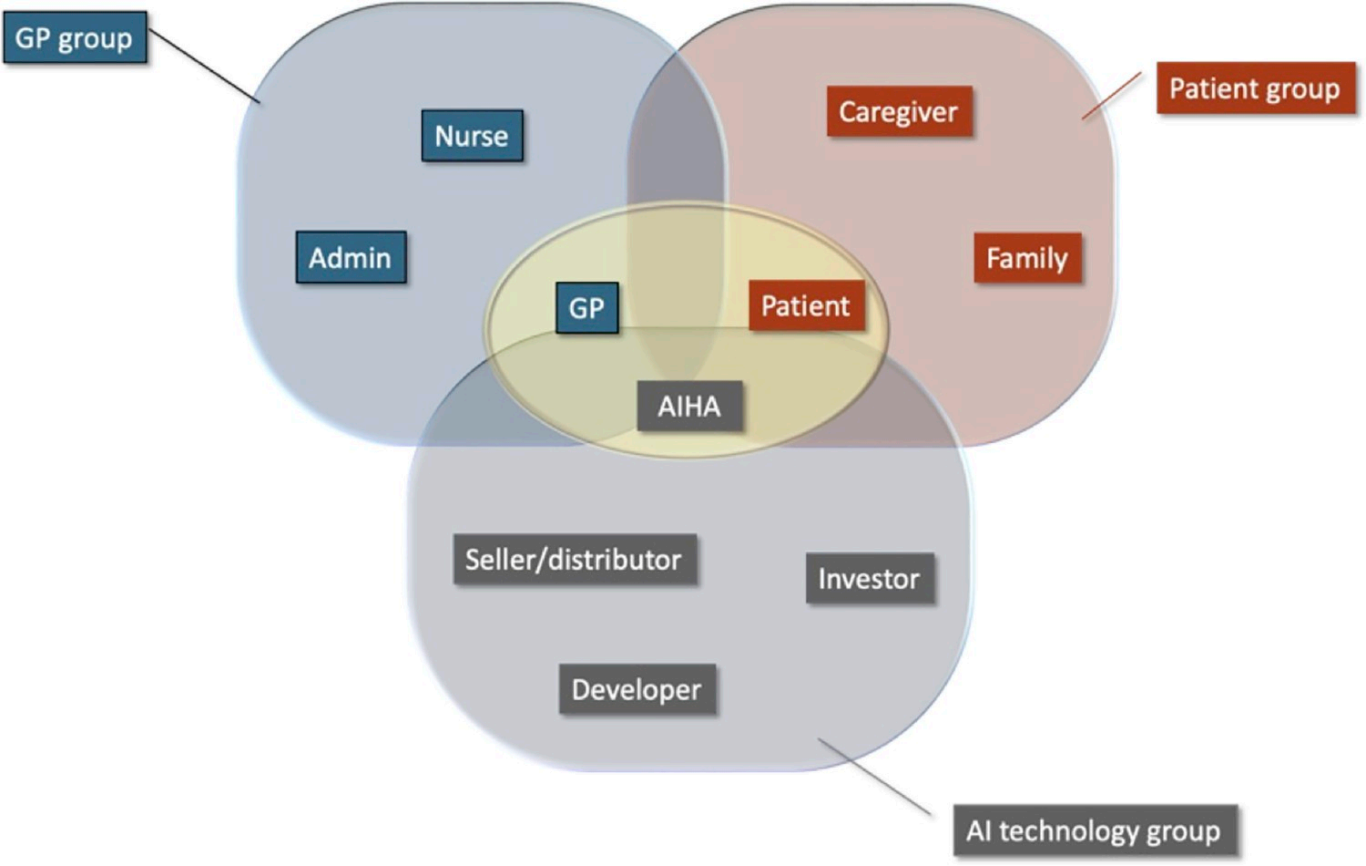
Stakeholders.

The researchers used their social and professional networks to recruit participants from three groups of stakeholders: Patient, GP, Tech (see Fig 1). The patient group includes patients, caregivers, and family. The GP group encompasses GPs, nurses, and administrative staff of a general practice clinic. The Tech group includes AI developer, data scientists, designers, technology salesperson. Participants were required to be 18 years or older, to speak English, and to reside in Australia. Participants in the tech group were exempt from the requirement to reside in Australia as an app may be destined for the Australian market, the technology could originate from another country. Participants were informed that the primary researcher (MG) was a PhD candidate, that the study was part of the requirements of her PhD. They were also informed that she has a background in computer science and worked in the high-tech industry for 25 years.

#### Data collection

**Fig 2:**
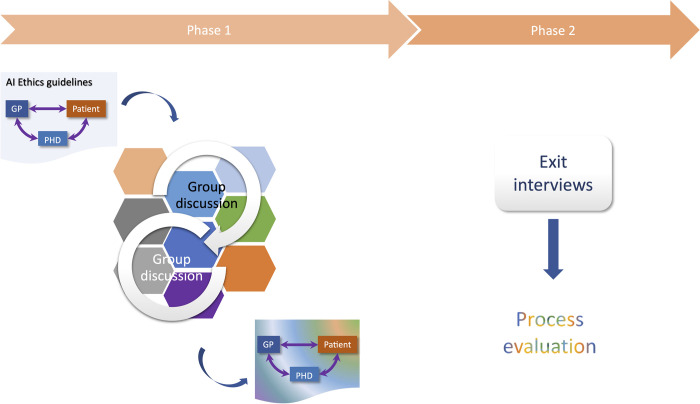
Two-phase study.

The study comprised two phases: 1) three 90-minute group conversations, 2) 30-minute semi-structured individual interviews (see Fig 2). Participants were sent a 7-minute video prior to the sessions describing the app, explaining what AI was and its limitations, and laying out the ethics guidelines from the Australian government [20]. Participants were asked to fill out a form capturing their demographics (see Appendix A–Demographics questionnaire in S1 Appendix). All sessions were run online. The group conversations were segmented in 90-minute-long sessions to facilitate sustained attention and enable scheduling flexibility. In the first session, participants were presented with a representation of the system of the app and asked to identify the information flowing between the different elements. The diagram was shared via an online whiteboarding tool, Mural.co, that allowed participants to take the pen if they wished to and add to the canvas. A series of questions based on CSH boundary critique were then examined (see Appendix B–CSH seed questions for group discussions in S1 Appendix). The process being iterative, each session started from the canvas built from the preceding session. At the end of each session, participants were asked to fill out a poll to capture their immediate feelings about the experience (see Appendix C–Workshop polling questions in S1 Appendix). In the individual interviews, participants were asked about their experience with consultative process and what could be done to improve it (see Appendix D–Semi-structured exit interview questions in S1 Appendix). The facilitator (MG) journaled her experience after each group session.

### Data analysis

Following a reflexive methodology, the analysis explored the data through multiple perspectives. Conversation groups and interviews were all conducted online and were transcribed using otter.ai. Both content and process of the study were the objects of analysis. Regarding the process, to evaluate the level of collaboration in the conversation groups, we identified threads of dialogue and the participants engaged in each thread and computed each participant’s airtime. To evaluate participants’ experience of the process, results of the polls at the end of each conversation group were aggregated and a thematic analysis was performed on the exit interviews, as well as on the main researcher (MG) journal entries. The analysis sought to answer the questions about what participants learned through the process, what could be done to improve it and make it sustainable. The outputs of the conversation groups, the diagram, and answers to CSH questions, captured in Mural.co were the basis for the content analysis, augmented with additional comments captured in exit interviews. The analysis consisted in clustering the vignettes of flow of information on the diagram and emerging themes from the clusters.

## Results

**Fig 3:**
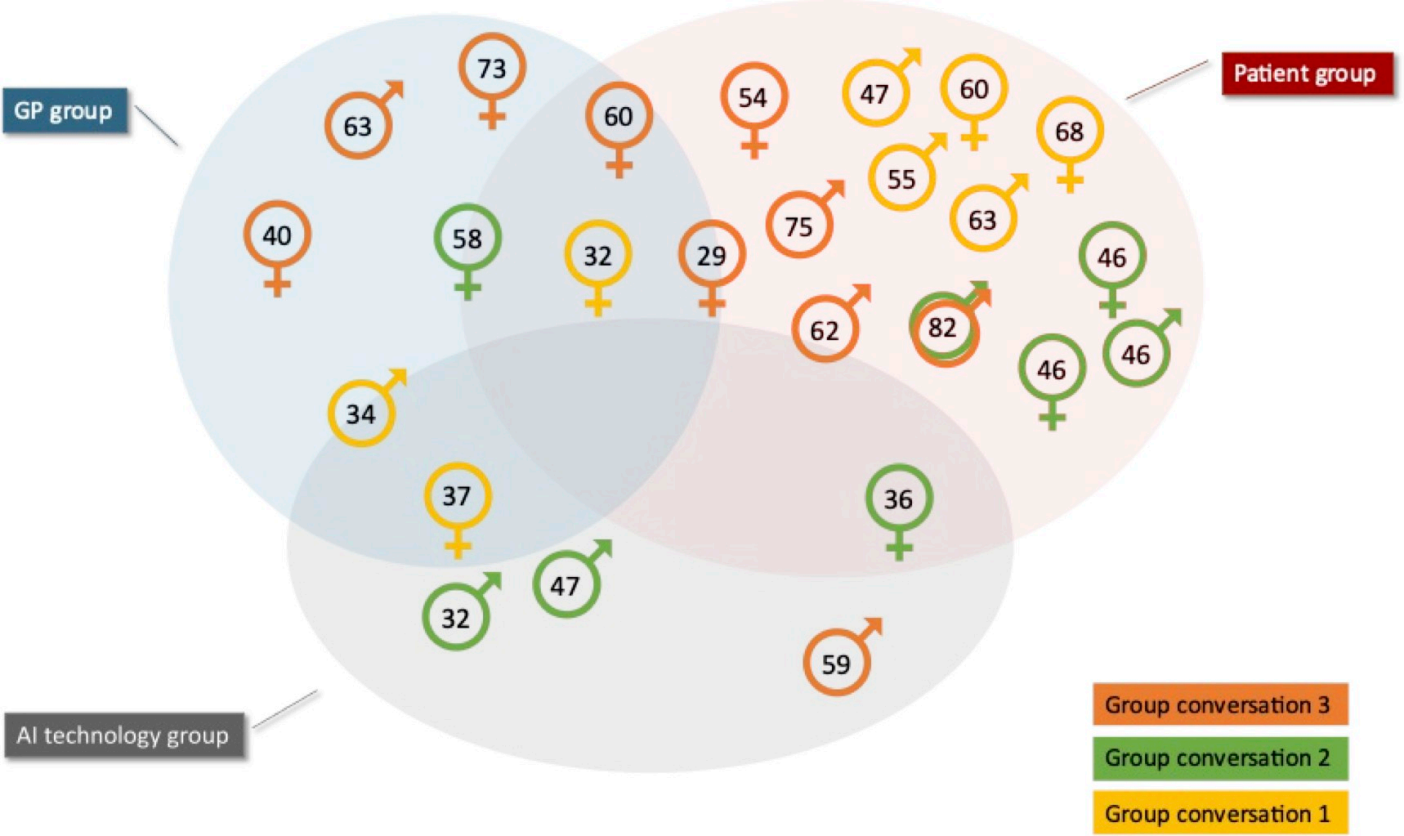
Group conversations demographics.

The first author (MG) conducted three conversation groups of 90-minutes between April 29 and May 5, 2022, and twenty-three 15 to 30-minute individual interviews between May 6 and May 13, 2022. While participants were invited to participate in up to three conversations, only one participated in two, and the rest participated in one only. Because some participants expressed an interest in following up with the output during their individual interviews, a group exit interview about the final model was held on June 14, 2022, which two participants joined. The final output of the four hours and thirty minutes of conversations is the collegial product of every participant. Another two participants were not individually interviewed as they did not respond to the follow up emails. Each stakeholder group was represented in every focus group, yet the second group had more tech than GP representatives which was inverted in the other two groups (see Fig 3). Some participants straddled two or more stakeholder groups. For example, one participant was a medical doctor and AI-based healthcare application developer thus represented GP and AI technology stakeholder groups. All conversations and interviews were conducted via Zoom online conference tool. Mural.co, an online collaborative whiteboard tool, was used in the conversation groups in addition to Zoom. Participants were invited to join Mural.co to be able to edit the whiteboard during the sessions. The whiteboard was also shared with participants in Zoom via the screen sharing functionality. All data were coded and analysed by the first author (MG).

### Participants

Twenty-five participants (12 males, 13 females) were recruited. Participants ranged from 29 to 82 years of age (M = 54 years, SD = 14.6). Participants provided written consent for inclusion in the study. Twenty-four participants currently reside in Australia, 12 live in urban centres, and 12 in semi-rural areas. One participant, from the AI technology group, resides overseas. The participants’ professional and cultural backgrounds were diverse, while all participants except two had a bachelor’s degree or higher level of education. The self-rated level of knowledge about AI ranged from no knowledge to expert programmer. See Table 1 for complete demographics of participants.

**Table 1:** Demographic distribution.

Gender	13 females, 12 males
Age	median = 54 years; span = 29–82 years; SD = 14.6 years
Location	12 in urban areas, 12 in rural areas, 1 overseas
Professional background represented	GP, nurse, remote health services manager, disability support worker, environmental health officer, social planner, human services designer, education, researcher, bio-medical engineer, data scientist, software engineer, I.T. consultant, agricultural consultant, lawyer, lecturer, administrator, strategy consultant
Cultural background represented	Anglo-Saxon, Chinese, Filipino, Bhutanese, European, Kurd, Persian, German-Peruvian
Highest level of education	1 Trade Certificate, 1 Diploma of Education, 4 Bachelors, 15 Masters, 4 PhDs
Technology savviness	The self-rated level of knowledge about AI is on average 4/7 ranging from 1 (no knowledge) to 7 (AI programmer) with a SD of 1.8

### How empowered do the participants feel and how sustainable is the process?

**Fig 4:**
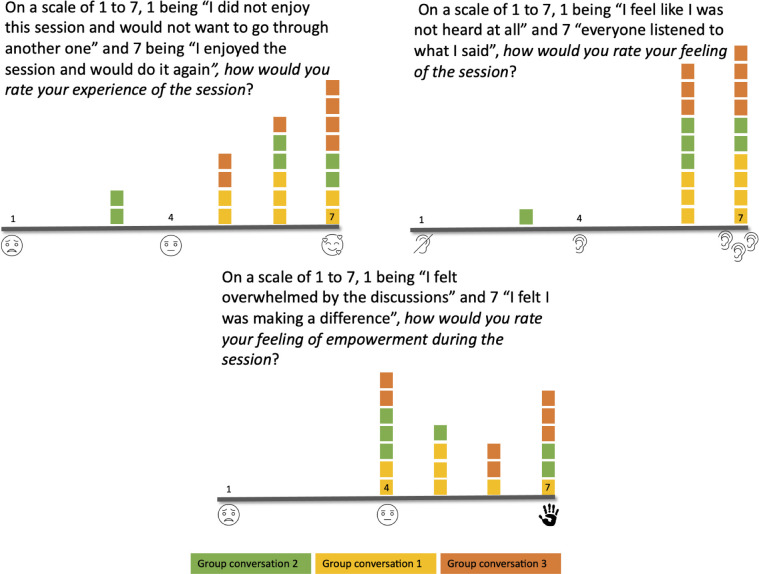
Polls results.

Seven out of eight participants for the first and second conversation groups, and seven out of ten for the third group responded to the anonymous exit poll. Most of the participants had a positive experience except for two in the second group (See Fig 4). Most participants felt heard during the conversations except for one in group two (See Fig 4). Seven participants felt neutral when asked whether they felt empowered by the session, while six felt they made a difference, with the remaining seven in between (See Fig 4). Being animated by a sense of purpose and understanding the impact of their participation were two drivers that could sustain the engagement of the participants in the participatory process. However, a perceived lack of expertise and fitness to take on the responsibility of the outcomes of the process were seen as barriers to a sustained engagement in the participatory process.

#### Sense of purpose as a driver to participation

Time was the most often invoked by participants as a barrier to contributing to a consultative process (GP/P3, Tech/P8, GP-Tech/P2, Patient/P5, Tech/P9). However, incentives such as recognition (GP/P3) whether financial or professional could alleviate the time factor. “Passion” (GP/P3) or “strong interest” (Tech/P9) in the topic were also nominated as a primary driver for participating in such conversations.

To support and sustain the interest, participants expressed a need to have clarity of the conversation’s objectives (GP/P18, Tech/P9, Patient-Tech/P14) and “for long term […] knowing how it can impact or help” (GP/P18).

GP/P21 sees the importance of participating in such conversations because “we know there are controls that are coming behind [AI in healthcare] that we feel powerless about even if we are working in the industry”, adding that the remedy to powerlessness is a

very coordinated and very strong response from clinicians themselves who provide the care, who are very ethically oriented, and those who really want to see the care of people and humanity managed well and not driven by corporations, private enterprise, and government.

#### Perceived lack of knowledge and responsibility as barriers to participation

A barrier to participating in a consultative process was to understand the level of responsibility when joining such a consultative process: “I have to […] know what my responsibilities are […] what are the implications of the system? And what are the risks related to that. So knowing those things […] would help me make a decision on participating” (Tech/P9).

Another barrier was the perceived lack of knowledge or expertise about AI (Patient/P5, GP/P3) because the conversation about some AI specific technical points can feel intimidating to the non-expert:

I didn’t feel like I had as much to contribute, especially with where the conversation was going, which was fine. But I just wasn’t sure on reflection, if I was the right person to be involved, or whether the group skewed so much to people who have such a like, like, considerable expertise in AI, that maybe it just very quickly moved from sort of a general conversation to quite specialized. And yeah, so that I guess that was my experience of it. I mean, I found it really interesting. And I think that all whole idea of it is very interesting. But as I said, I don’t know that much about it. So it did make me think, oh, like maybe I’m not like the best person to be commenting so much. Or if I am maybe I need to be like in the beginners’ group or something (GP/P3)

### How is collaboration enacted?

In the group conversations, participants engaged in dialogues that were respectful of each other’s voice, and inclusive. During these dialogues, stories and expertise were shared, and the model got enriched and expanded from these exchanges. The diversity of the experiences shared contributed to participants’ better understanding of others’ point of view. However, diversity of backgrounds and the openness of the conversation boundaries made it more challenging for some to follow the different threads of conversation. Yet the discomfort felt by some was also deemed to stimulate thinking.

#### Dialogues

**Fig 5:**
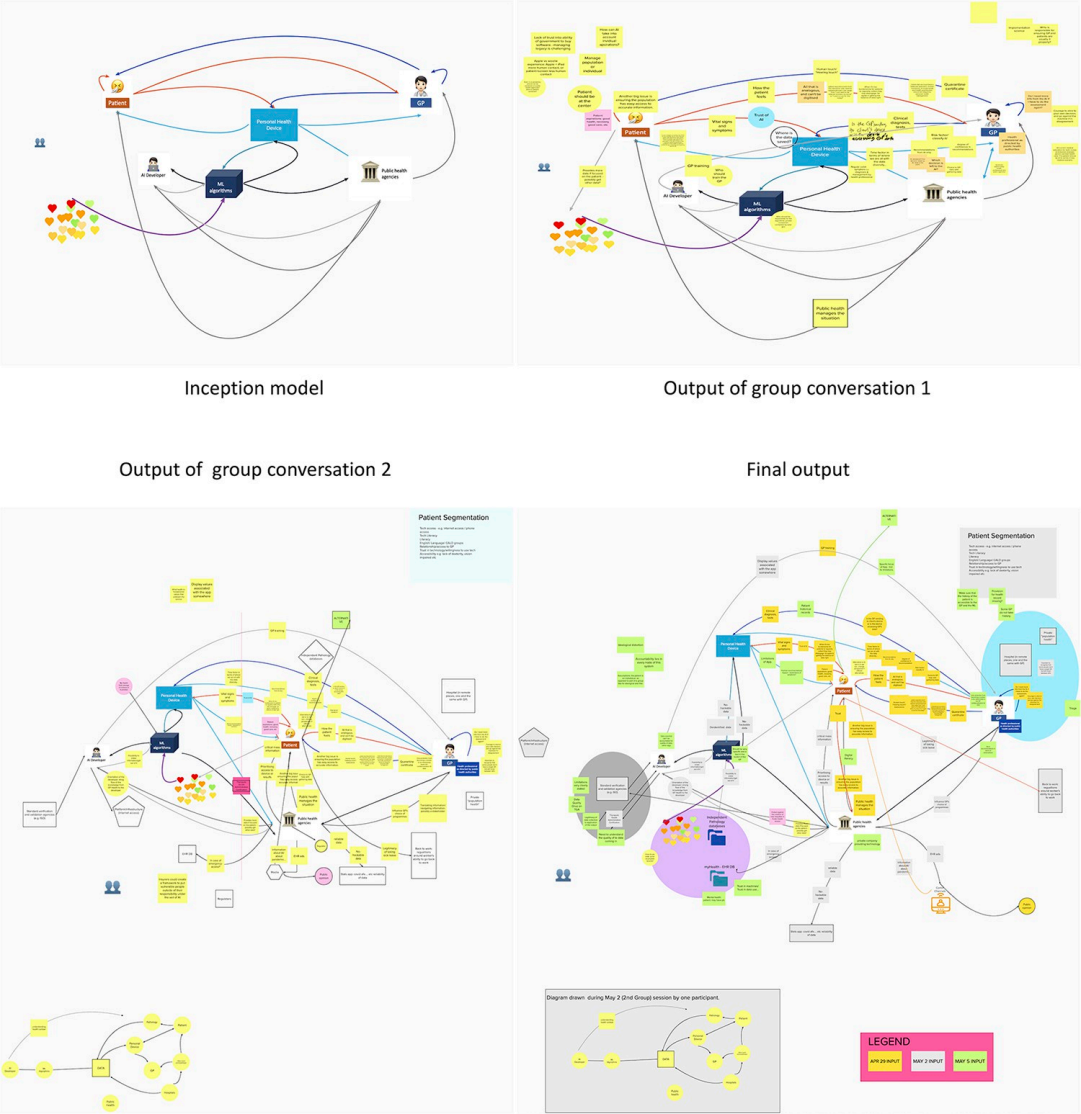
History of conversation group outputs.

The participants in the first conversation were presented with a diagram representing the system GP-Patient-AIHA with no further information while the second and third group built on the output of the previous group (see Fig 5) (full canvas available in Appendix E in S1 Appendix). While all participants were invited to join Mural.co and possibly edit it, the canvas was also shared through the screen sharing functionality in Zoom. Four participants in the conversation group two (Patient/P10, Patient/P11, Patient/P12, Tech/P15) and one in conversation group three (Patient/P10) took the pen. In each conversation group, participants from the GP-Tech and Tech group, that is with the most expertise, used the most airtime (see Table 2). The threads identified in the conversations involved dialogues between two to seven participants, with a few involving only one participant. The latter were reflections or ideas that spurred no further development in the discussion. As such dialogues were inclusive which is reflected in the polls as most respondents (twenty out of twenty-one) reported a feeling of having been heard (Fig 4). However, Patient/P11 reflected that people taking the pen in Mural created some monologues.

**Table 2:** Airtime distribution per participant per conversation group.

FG	Stakeholders	Speaker	Gender	Age	AI literacy	Airtime	Median and average	Took the pen	Number of threads participated in	Total number of threads
1	GP-Patient	P3	Female	32	2	2:23	5:58 6:42		2	11
1	Patient	P5	Female	68	1	2:43			3	
1	Patient	P6	Female	60	3	5:20			3	
1	Patient	P7	Male	55	3	5:55			3	
1	Patient	P4	Male	47	4	6:02			5	
1	Patient	P1	Male	63	5	7:49		Added notes	3	
1	GP-Tech	P8	Female	37	7	8:36			4	
1	GP-Tech	P2	Male	34	6	14:51			7	
2	Tech	P9	Male	32	6	1:36	5:32 6:10		1	16
2	Patient	P12	Female	46	4	3:56		Drew another version of the diagram that was data-centric	4	
2	Patient	P16	Female	46	4	4:06			2	
2	GP	P13	Female	58	4	5:07			3	
2	Tech-Patient	P14	Female	36	4	5:58			4	
2	Patient	P11	Male	46	1	8:22		Added notes	4	
2	Patient	P10	Male	82	3	9:11		Added notes	7	
2	Tech	P15	Male	47	7	11:06		Added notes	6	
3	GP	P18	Female	40	2	1:45	3:46 5:11		2	19
3	Patient	P22	Male	75	5	2:25			3	
3	Patient	P24	Male	62	5	2:40			3	
3	Patient	P25	Female	29	5	3:09			2	
3	GP	P23	Female	73	4	3:44			4	
3	Patient	P20	Female	54	1	3:48			3	
3	GP	P17	Male	63	3	5:07			3	
3	Patient	P10	Male	82	3	6:05		Added notes	4	
3	GP-Patient	P21	Female	60	5	6:15			4	
3	Tech	P19	Male	59	7	16:56			9	

#### Knowledge exchange

Knowledge transfer happened during the conversations. For example, GP/P18 related to have gained a better understanding of how data is processed to train an AIHA from Tech/P19 who shared his technical knowledge and experience as a data scientist in simple terms using metaphors:

well think of think of a data scientist like a Cuisinart, right? I mean, a data scientist is a Cuisinart. Whatever comes out of the Cuisinart is only going to be as good as the vegetables that you put in the Cuisinart. Like, blender or something. Yeah, right. In other words, it’s stupid. A blender is stupid. It doesn’t, but it knows how to blend really well. Right. So it requires a cook or a chef to make sure you put in the so it really requires medical professionals to oversee the whole process at every step.

GP/P23 shared that she gained a new way of thinking about AI rather than new learning. Personal experiences related to COVID-19 or to an AIHA were shared. For example, Patient/P1 shared his experience with the NSW Health Services when he contracted COVID-19 which helped identify additional streams of information such as direct links between patients and health agencies.

#### Model is expanded and enriched

Additional stakeholders originally not on the map emerged from the dialogues, notably the media, and other sources of information for the public. The original representation of the system had the AIHA in the middle. The first conversation, with more representatives of the GP group than tech, brought the idea that it should be patient centred. The diagram was adjusted between conversation one and two to reflect the wishes of the group. In the second conversation, in a group with strong Tech representatives, the data were seen as the centre of the system. Interestingly, a participant (Tech/P19) of the third conversation in his exit interview remarked that the AI is spread throughout the stakeholders and as such is not centre, but rather distributed.

#### Diversity of participants pluses and minuses

The diversity of backgrounds among the participants in the conversation helped them develop a better understanding of other points of view and needs. GP-Tech/P2 found the conversation interesting “especially some of the things patients said about […] their experience, and how they felt about […] the algorithm making decisions, or past experiences with healthcare”. GP/P18 reflected on the differences between her experience practicing in an urban area and the stories shared by a participant (Patient/P20) from a rural area where there is not enough GPs:

I can quite comfortably go, I wouldn’t see a patient, I won’t see new patients, because I know they’ll find someone else there. [rural GPs] probably can’t, but it ends up that […] they just can’t know their patients. And they’re just going okay you come up with this problem. I’ll fix that problem like this, but that’s all I can do. I’m just keeping you alive.

Patient/P10 reported how listening about stories from remote areas was

quite a new thought for me of the possibilities, but also the problems of doing that sort of thing, and the dependencies it can create then for the second and third order consequences […] had my eyes opened on a number of issues.

GP-Patient/P21 reflecting on the different degrees of understanding of AI among potential users and the consequences of these variations concluded that having conversations such as the ones in the study was desirable:

when looking at the perspective of people who have not got to that point, in terms of thinking about [AI in healthcare], and but already raising the things about ‘well what’s it going to mean for me as a patient?’ What’s going to be, mean for me and for me, who’s not maybe technically as advanced as the Yeah, and I thought about it Well, I was I wished I had the knowledge of the [data scientist participating in the conversation], because I probably would feel more comfortable. So these things are worrying and I thought it was good, diverse group for us to think about those issues.

However, Tech/P9 wished for more homogeneity among participants’ background because he found:

hard to follow other people’s opinions […] each person wanted to talk about their own specialty and their own experience, and sometimes […] it was hard to follow […] I didn’t exactly understand what they were talking about […] if these people were from similar backgrounds, maybe it would have been more manageable.

Most participants reported appreciating the visual model, in particular the ones with a technical background related that they were familiar with such imagery. GP-Patient/P17 though reflected how he preferred stories, and the visual support did not really add value to him. Some in the Tech group (Tech/P8, GP-Tech/P2, Patient-Tech/P14) wished for more focus on certain points, stronger boundaries in the conversations. Patient/P11 noted the number of streams of thought felt at times difficult to follow, while at the same time highlighted the rich number of points of view present in the groups. Patient-Tech/P14 reflected that being confronted with different perspectives while possibly jarring stimulated her thinking.

### What improvements to the ethics framework upon which the app design is based are brought forth by the collaborative process? How is ethics instilled in the design of the app? Who are the custodians of the process?

The content output is what has been captured in the diagram (See Fig 5) during group conversations. By design, each piece of information is not attached to a participant but should be considered as the product of dialogues among participants. The enrichment of the model included (1) an expansion of the stakeholders, and (2) distinction between explicit and implicit or subconscious information and knowledge that is flowing through the system. Other themes were (3) enablers for receiving or giving information which is about the readiness of the different agents to receive and give the identified flow of information and knowledge, (4) circles of influence that is the ethics of influence of some agents of the system over others, (5) risks associated with data breaches, (6) the transparency of values embedded in the system, (7) primary and secondary purposes, (8) second and third order consequences, and (9) custodianship.

#### Expansion of the stakeholders

The GP was extended to encompass hospitals, as in remote areas of Australia they are one and the same. Communications channels such as media outlets, social media were identified as playing a key role in informing the public but also shaping the attitude of patients and clinicians towards the AIHA, AI in general, or the organisation behind the AIHA. Organisations managing health records, whether private, such as independent pathology services, or governmental, such as the My Health Record initiative in Australia, were added as the principal agent between AIHA and health agencies. Regulators such as the Therapeutic Goods Administration (TGA) which is the equivalent of the American Food and Drug Administration (FDA), were added as the go-between for the AI developer. Infrastructure such as internet services was also added because it is the foundation for the system to function.

#### Explicit and implicit or subconscious information and knowledge

Two different types of information emerged from the diagram: (1) the kind that is captured in electronic health records or by sensors such as vital signs or symptoms, (2) the kind that is qualitative in nature and conveys feelings, expectations, attitudes, values, or gestures. The latter included “healing touch”, “reassurance” and is prominent in the relationship between the GP and the patient. The contrast between the two types of information elicited concerns and expectations. For example, the relationship between the patient and the GP was seen as the “only protection from becoming a number”, which pertains to the dignity and agency of the patient. GP/P18 noted that because she knows a patient, she can pick up if something is missing or possibly wrong in the health record which is tied to accountability issues and autonomy of the GP.

GP/P23 observed that EHRs hold objective as well as subjective data. On the other hand, the importance of health records was acknowledged to facilitate care across different clinicians or services which is linked to safety.

#### Enablers for receiving or giving information

Communicating the parameters of the information delivered by the AIHA was given importance. Information such as “time factor in terms of where we are at with data diversity”, “risk factors”, “degree of confidence in the recommendation” issued by the AI were identified as needing to flow from the AI to the GP. It was also noted that the GP needs to be able to stick to his/her own decision if in disagreement with the AI. “Limitations of the app”, “practical recommendations, support, explanations of symptoms” were identified to flow from the AI to the patient. “Digital literacy” was suggested to flow from health agencies to patients. These concerns are related to ethics principles of autonomy, explainability, transparency and responsibility both for patients and GPs, and fairness as in inclusiveness and accessibility.

#### Circles of influence

Some information and knowledge identified in the conversations could be constraining or influencing the recipient by design. As emerged from the conversation, health agencies influenced and possibly restricted GP’s choice of treatments through their flow of information. The media emerged from the conversations as an agent channelling information to the public carrying significant weight on the behaviours and attitudes of members of the public and health professionals towards the adoption and trust of the AIHA.

Potential sources of deception identified by participants were advertising and marketing of AI and the AIHA. To preclude or lessen deception, it was suggested to make processes and information transparent, and accessible to the public. For example, where the data used to train the AIHA, and collected by the AIHA is stored and the level of deidentification of the stored data should be public knowledge and made transparent. It was also suggested that differential privacy, that is systematic deidentification of the data, should be the default for data processing. Another suggestion to preclude or lessen deception was to increase AI literacy of clinicians. Another suggestion was to limit the amount of collected data seemingly unrelated to the application.

These flows are characterised by trust and have implications about the autonomy of the recipients.

#### Risk associated with data breach

Concerns about information flows being hacked, misused, or repurposed were discussed. Hacking of data, breach of privacy happening between the AIHA and other agents such as health agencies, patients or GPs was brought to the fore. It was suggested to ponder the consequences of a malicious usage of the data and weigh in whether the benefits of the AIHA justifies the risk.

#### Transparency of the embedded values

Beyond the boundaries within which the AIHA operates, such as its limitations and potential biases, it was suggested that the values upon which the AIHA are built should be communicated to the GP. It was also noted that an assumption made by the whole system was that the patient is treated as an individual, and not as part of a larger group, the latter being an aboriginal view for instance.

#### Second and third order consequences

An important consideration that arose in the discussions was about people who would or could not be part of the system under consideration, that is not having to use the AIHA. The key questions that arose from this thread were: What is the alternative for people not able or willing to participate? Could it affect their ability to work? Would there be a compensation system if it is the case?

The noted challenge with AI is that the performance of the programme relies on the quality, quantity and diversity of the data and leaving out parts of the population weakens the whole programme and could further entrench inequalities and accessibility. Another option was to have the possibility to opt out one’s health data from the circulation of information or contributing one’s health data in case of emergency such as the pandemic.

Another consideration was the environmental cost of the whole system in terms of carbon footprint of the required devices, storage, bandwidth etc. Importantly, it was reckoned that ‘what health is’ should be part of the foundations of the whole, and a question that needs to be considered is whether such an app could lead to viewing health through a narrow slice of data. It was suggested to keep track of the shadow of the system, that is the negative fall outs.

#### Primary and secondary purposes

The original purpose of the app was stated as enabling GPs to make better more informed decisions about how to treat COVID-19 and keeping clinicians safe. The following purposes were added: decreasing the workload of GPs during the pandemic, empower patients to manage COVID-19 related issues, limit the disease spread, and for the AIHA developer to increase revenue. The primary beneficiaries identified were clinicians, and patients. Additional beneficiaries identified were health insurance agencies, population at large, and astutely the people who are represented in the dataset used to train the AIHA. To know whether the purpose had been achieved, participants suggested assessing how protected clinicians are from getting infected and evaluating the feeling of isolation and self-confidence of COVID-19 patients.

#### Custodianship

The questions about who can guarantee that the AIHA system abides by its intended purpose and meet ethical standards, and how it can be guaranteed have been addressed at different levels. A need for an independent auditing process of the data and algorithm was identified during the conversation groups. The Therapeutic Goods Administration (TGA) was suggested to meet that need. Another suggestion was to that there should be a Chief Patient Officer from the organisation owning the AIHA held responsible for all outputs of the AIHA. A data scientist participant (Tech/P19) acknowledged that data scientists need domain expertise knowledge to interpret data correctly and would not want to bear the responsibility for it. Another complexity lies in the different agents involved with data acquisition and storage when one follows the flow of knowledge: AIHA development organisation, health agencies, as well as EHR repositories, whether government owned or private such as private pathology databases. Standard validation and verification agencies, such as ISO for example, were identified as part of the solution to clearly state the requirements of data quality. Transparency of the processes and data collected was also invoked as a mechanism to ensure that the AIHA is serving its intended purpose.

At the user level, it was deemed necessary to have the clinician in charge of the final decision, implying that the AIHA should not be an automated decision-making system. Yet, it was remarked that if there is no decision made by the AIHA, it could be a superfluous system.

If the system does not prove or stops to serve its intended purpose It was suggested that users should be able to opt out of the system and have their data removed from the system. It was also suggested to keep track of the shadow of the system, its negative impact as a trigger to possibly put a halt to the whole service.

Knowledge and experience identified during the group conversations that should contribute to the AIHA implementation to guarantee it meets its intended purpose and meet ethical standards were implementation science, user experience design, health equity research and advocacy, medical expertise to help data scientists with data quality, complex health condition care coordination, experience of the AIHA users from diverse backgrounds such as non-English speakers. It was also suggested that other desirable sources of knowledge could come from including an archetype of the AIHA, and enlisting the help of artists, writers, or mythology experts.

## Discussion

In this study, we set up a consultative process with a range of stakeholders looking at the flow of knowledge between the different agents of an AIHA. We sought to investigate the outcomes but also the process of the participatory engagement. We sought to understand the collaborative process between the participants, and the drivers of their participation. We also sought to explore how ethics principles were implemented in the AIHA and the possible improvements or refinement of the ethics framework brought by the participatory process, and how to guarantee the implementation of the ethics framework. We identified three factors in the participatory engagement. First, clarity of purpose and transparency about the impact of their contributions are key drivers to participants’ engagement and sustainability of the process. Second, technology literacy of the participants affected both positively and negatively their ability and willingness to partake into the conversations. Third, the model and process anchored in SSM principles supported dialogues among participants and allowed for a collaborative experiential knowledge exchange which created a learning opportunity for the participants. Four guidelines came from the investigation of the outcomes. First, ethics happen within the relationships between the agents of the system, and the relationships to be considered extend beyond the ones stemming from the AIHA. Second, transparency should extend to the values embedded in the AIHA and all processes including ethics implementation. Third, there should be an examination of the alignment of the stated purpose of the app with the purposes of the main stakeholders. Fourth, there is a need for an independent organisation to oversee the custodianship of the data and the process, including the implementation of ethics.

### Process 1: Clarity of purpose and transparency about impact of contribution are drivers for participation

As expressed by participants, a sense of purpose, and clarity about the impact of their participation are key to their engagement. A corollary is to ensure that they are able and willing to take on such a responsibility. It raises ethical questions for the instigator and facilitator of the process: Can the participation in such conversations put too much of a burden on the participants because of the perceived responsibility? While candid contributions are desirable in Systems Thinking to avoid groupthink, can the participation into the process put some participants who don’t think they have the knowledge or feel intimidated by the whole topic in a stressful position? This could be mitigated by providing regular reassurance to the group that all voices are equal regardless of AI literacy. Because the process itself is educational, it is also possible that participants confidence would build over time. However, it means the instigator and facilitator of the participatory process bear the ethical responsibility of the readiness of the participants to engage in the process.

### Process 2: Considerations about technology literacy

From an earlier study (see S1 File), it is assumed that such a consultative process should happen at the inception, or design time of an AIHA, but should also be sustained throughout the lifespan of the application because of the ever changing, evolving nature of the technology, societal values, and perception of the technology. In a Systems Thinking driven participatory process, diversity of background is important, and includes stakeholders who do not qualify as experts. Yet, because AI is perceived as complex, even participants with expertise in related fields to the GP-Patient-AIHA system, but no AI knowledge, can feel inadequate and inhibited from sharing their experience. Dialogues were inclusive and all participants were given a voice. However, the usage of an online whiteboard tool offered the technology savvy participants an additional channel of contribution while others were restricted to the verbal conversation. Additionally, participants with AI expertise tended to use more airtime. It suggests that technology literacy can facilitate but also distract from conversations with other participants. On the other hand, participants reported having learned from the AI experts, and some reported having evolved their thinking about medical AI and its ethics because of the conversations. Such a forum offered an opportunity for participants to share their assumptions and check how they fare in relation to other participants with a deeper knowledge of one aspect of the issue than theirs.

### Process 3: Dialogues supported by the model are effective at surfacing assumptions

Familiarity with the type of visual representation used in the model (Fig 5), which is common in technology circles and was created by the main researcher (MG) who also had a technical background, eased the conversation for some. The model got expanded and reframed throughout the process by the different groups. Notably, the discussion about what should be at the centre of the diagram, the patient, the data, or the GP exemplified the worldviews held by the different participants. From a systems thinking perspective, the model is only a representation of the system and assumed to be incomplete and imperfect but designed to steer conversations [21]. Allowing for such rearrangement by the group is a way of surfacing some assumptions, or preconceived ideas.

Framing the discussion around the flow of information and knowledge being exchanged between the GP, the patients, and the app, the developer, and the health agencies led to the inclusion of additional stakeholders such as the media, and led to ethical discussions about sources, custodians and broadcasters of the information and knowledge. It also led participants to share stories. As such, the process allowed for free flow of knowledge between participants, the model used helped surface assumptions, and values, and created a richer picture of the GP-Patient-AIHA system over the course of three conversation groups in an online format. If the process could be held in a physical format, possible improvements to the process would be to include a kinaesthetic presential component to engage more senses, remove some technology literacy barriers, be more inclusive of indigenous traditions as explored by some systems thinkers [14,22].

### Outcomes 1: Ethics happen in relationships including the ones on the fringe

Asking to identify information and knowledge flowing throughout the GP-Patient-AIHA system induced reflections about what is passing through or exchanged in the relationships between the agents of the system, where it comes from, who controls it, what the constraints of the exchange are, and led to the expansion of the network of relationships. As such it revealed ethical issues arising in the relationships within the broader system, not only the relationships directly linked to the AIHA such as the one with the media for example. It implies that implementing ethics in an AIHA needs to consider relationships beyond the immediate ones stemming from the AIHA. It also implies that implementing ethics needs to happen within the relationships, which involves enabling agents for such a relationship to be ethical. This reinforces the findings of a prior study (see S1 File) that found that ethical issues arise within the relationships, and because the autonomy and sense of responsibility of the consumer of information are affected by the way the information is delivered, enabling consumers of information to be able to receive the information is part of the ethics.

### Outcomes 2: Transparency of values and processes to augment the ethics framework

The distinction between explicit and implicit, or hard and soft information/knowledge exchanged between agents made by participants has implications. Hard and soft to qualify knowledge seemed to be apt in reference to hard and soft skills which originally were distinguished by whether the skill involved a machine (hard) or not (soft). The soft knowledge was valued, values-laden, and seen as a safeguard against what could possibly go wrong with the use of an AIHA including being assimilated as a number. It could be argued that it is similar to the self-driving car conundrum where even if the AI is safer than the human drivers, the people are not ready for it. Beyond the safety debate, from an ethics point of view, the values embedded in the soft knowledge are human specific and assumed not to be able to be produced by a machine. Questions to examine is how the hard knowledge and the soft knowledge work together: Do they reinforce each other? Do they conflict with one another? While the Australian AI ethics guidelines mentions how human oversight should be enabled to ensure accountability [20], having the soft and hard knowledge work in harmony implies that the AIHA enables or does not disable human to human relationships. Another question to examine then is: Does the AIHA impede the relationships between humans? Further, the values embedded in the system should be made transparent and communicated clearly which includes biases of the training set for example, or what are the risks associated such as in the case of a data breach? As suggested, mapping the shadow of the system, and communicating it would also be part of implementing ethics. The shadow would include second and third order consequences such as the environmental and social negative impact of the AIHA. Hence, the principle of transparency encompasses transparency of processes, and of data usage and custodianship, and of the shadow of the system.

### Outcomes 3: Alignment of subsystem’s purposes with stated purpose

The fifth guideline of the Australian AI ethics framework [21] about reliability and safety recommends that the AI “operates in accordance with the stated purpose”. As emerged from the conversations, the originally stated purpose which was GP centred was revisited and augmented with patient-centred purposes. It could be seen as stated purpose compared to purpose as expected or imagined by consumers. The latter could be considered as subsystems purposes. It is then required to ensure that they align with the overall purpose. One limitation of the study is the absence of financial considerations and stakeholders from the conversations which could have emerged problems of alignments within the subsystem’s purposes. For the fifth guideline to be implemented, it then means to have transparency of the purposes of the subsystems and understand how they align with the stated purpose.

### Outcomes 4: Independent custodianship of the process and the data

There is a need for an independent organisation to be the guarantor of the quality of the algorithm and data of the AIHA in terms of ethics. It seems to align with the concept of “ethics as a service” developed by Morley et al. [23]. The TGA and organisations such as ISO were advanced as guarantors. Complementary schemes were suggested in a prior study (see S1 File) which would involve an organisation independent from government and private interests. It echoes lines of reflection from other researchers in the field advocating that “[f]or technology to truly work in the public interest, we need to invest in building organizations that are free from corporate profit motives and that respect, integrate, and compensate communities with whom they work” [24]. While defining the exact structure of such an organisation goes beyond the scope of this paper, this is a line of enquiry that would need to be pursued. One challenge of medical AI ethics implementation regarding health data collections and curation is the distributed network of agents involved. As highlighted in the results, another complexity is the range of expertise required which calls for a transdisciplinary approach, of which the participatory process of this study is a reflection. Also reflexively, because the facilitator of the participatory process can influence outcomes and has an ethical responsibility, it means that participatory processes should be facilitated by the same kind of independent body.

### Further considerations

Important questions to provision for when implementing ethics is: Who can make the decision to halt the programme if deemed unfit of the intended purpose? How do we know it is unfit? While this study does not answer the first question, monitoring the shadow of the system, that is its negative impact should be part of the information needed to know when the programme is unfit to the intended purpose.

### Limitations

This study included the voices of 25 participants from diverse backgrounds yet lacked representation of the younger generation as the youngest participant was 29. Most of the participants had some tertiary education degree. With technology becoming so entrenched in everyday life from an increasingly younger age, it is critical that future research look to understand the perspective of younger people in medical AI. Another limitation of this study is the absence of financial interest representation. For one, it is a fictitious AIHA and there were no participants with an investor or chief financial officer’s profile. Given the role of money in shaping behaviours, it is critical that future research explore the perspectives of investors and finance when implementing ethics. Finally, the data collection and analysis were performed by one researcher limiting the ability to avoid colouring findings with the researcher’s biases. To address this limitation, the adopted reflexive methodology that includes reflective journaling of the one researcher helped make biases transparent. Notably, this limitation is inherent to any participatory process and reinforces the need for reflexive methodology.

### Recommendations & conclusion

Because of the complexity of Implementing ethics in an AI Healthcare Application, a systemic intervention based on Soft Systems Methodology and Critical Systems Heuristics is well adapted to the endeavour. Soft Systems Methodology and Critical Systems Heuristics involves sweeping in experiential knowledge from diverse stakeholders through a participatory process inclusive of diverse and dissenting stakeholders. The consultation process should be employed at the inception of the design of the app and carried on regularly with any update. Consultation should be an integral part of the app lifecycle and should involve clinicians, patients, and AI technology representatives from diverse cultural and professional backgrounds including stakeholders that are on the margins of the groups. As such, the ethics implementation process should consider the following: (1) To sustain the engagement of the stakeholders into the participatory process, it is important to communicate why they are invited to participate and what their responsibilities and possible liabilities are. The facilitators are the custodians of the participatory process and responsible for maintaining an egalitarian engagement. (2) It is important to reassure participants that all voices are equal regardless of their expertise in the field of medical AI. (3) Using a visual representation of the AIHA and asking participants to map the flow of knowledge between agents in the model is effective to surface assumptions and facilitate experiential knowledge and stories sharing. It is to be expected and desirable that the model will evolve along the conversations shifting focus and expanding its network of relationships. (4) There is a need to examine how ready the different agents in the system are to receive or give the knowledge flowing to or from them. (5) The dynamics of the relationships matters. When examining the flow of knowledge, it is important to distinguish between hard knowledge (digitised information) and soft knowledge (all that is not machine conveyed) and examine the dynamics between the two and whether one is reinforced or impeded by the other. Other questions flowing from this thread are how does the AIHA affect the relationships between humans? How is the oversight of the AIHA by humans enabled? (6) Transparency of the values and processes needs to be considered. A question to examine is how are the values embedded in the AIHA communicated and made transparent? (7) There is a need to check the alignment of the purpose of the system as stated with the purposes as expected or imagined by stakeholders and explore how to correct any misalignments. As such, stakeholders need to express what they expect the purpose to be. (8) The custodianship of the data and algorithm, and the process of implementing ethics needs to be made transparent. Ideally, the custodians should belong to an independent organisation. Additionally, one question that should be addressed at inception and revisited regularly is who can make the decision to halt the programme if deemed unfit of the intended purpose?

Presently, the choice of how to implement ethics remains in the hands of the organisation designing the AIHA, or the sponsors of the app. Some organisations choose to implement ethics to create a fair and safe application, while others are motivated to do so to increase the uptake of the application. The approach proposed in this study is a systemic intervention whose skills and competencies could be developed by the AIHA sponsor organisation or outsourced to an organisation with these skills and competencies. Ultimately, the *motivation* for adopting the approach recommended in this study matters and would make the difference when it comes to choosing between doing the right thing and doing things right.

## Supporting information

S1 Appendix Appendices include questionnaires used in the conversation groups, and the output of the conversation groups. (DOCX)

S1 File Prior study. Prior study data. Data from a prior study upon which this study is built.
(DOCX)
