# Supplementary material for: Mapping the flow of knowledge as guidance for ethics implementation in medical AI: A qualitative study
Source: PLoS One. 2023 Nov 2;18(11):e0288448. doi: 10.1371/journal.pone.0288448 (PMC10621848; doi:10.1371/journal.pone.0288448)
Supplement: S1 Appendix — (DOCX) [file pone.0288448.s001.docx]

Appendices

Appendix A - Demographics questionnaire

1. What gender do you identify with?
   1. Male
   2. Female
   3. Other
   4. Prefer not to say
2. How old are you?
3. What is your postcode?
4. What is your current occupation?
5. Which occupation have you spent the longest time practicing in?
6. What is the highest degree or level of education you have completed?
7. What is your cultural background or heritage?
8. How would you rate your knowledge of Artificial Intelligence on a scale from 1 to 7, 1 being I don’t know much about it, and 7, I have worked with AI software?


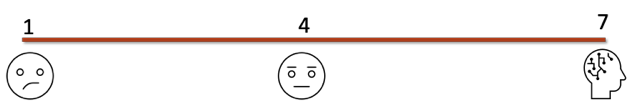


Appendix B – CSH seed questions for group discussions

*Motivation*

1- Who should be (or is) the beneficiary of the app? and how should or do they benefit from it?

2- What is or should be the purpose(s) of the app? (Tied to this question are the consequences - which should lead to the what ifs questions to identify possible unintended consequences)

3- What is or should be the measures of success of the app? (In healthcare, it involves an evidence-based approach to the benefice of the intervention/service)

*Control*

1- What is or should be the decision-making process concerning beneficiaries, purposes, measures of success (motivation)?

2- What are or should be the resources, means, policies etc that should be under the authority of the decision makers?

3- What is or should be essential (the environment, the conditions) to achieve the benefits and purposes but should NOT be under the authority of the decision makers/making process?

*Knowledge/expertise*

1- Who are or should be the experts involved (in addition to or instead of the decision makers) in delivering the benefits?

2- What counts or should be counted as expertise and in what capacity/role? (In AI, this could be also an AI?)

3- Who or what should or would guarantee or increase the likelihood of success? (Could be political, social, or consensus or...)

*Legitimisation*

1- Who represent or should represent the interests of the affected including the ones without a voice (includes unborn, non-human.)

2- To what extent the affected are able or should be able to opt out, not to participate, or be enrolled?

3- What core values, assumptions, worldviews should be or are considered, and should or underpin the app/service and how to reconcile them?

Appendix C – Workshop polling questions

1. On a scale of 1 to 7, 1 being “I did not enjoy this session and would not want to go through another one” and 7 being “I enjoyed the session and would do it again*”, how would you rate your experience of the session*?


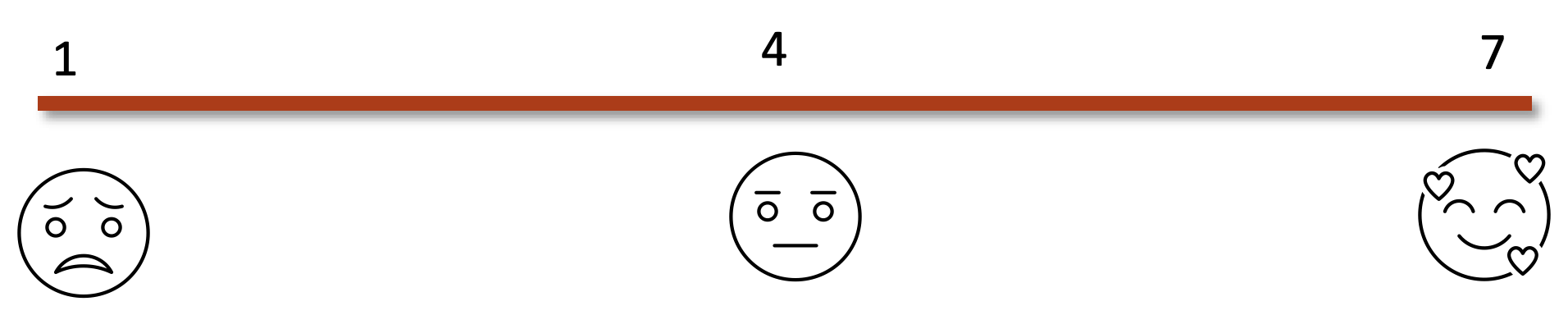


1. On a scale of 1 to 7, 1 being “I feel like I was not heard at all” and 7 “everyone listened to what I said”, *how would you rate your feeling of the session*?


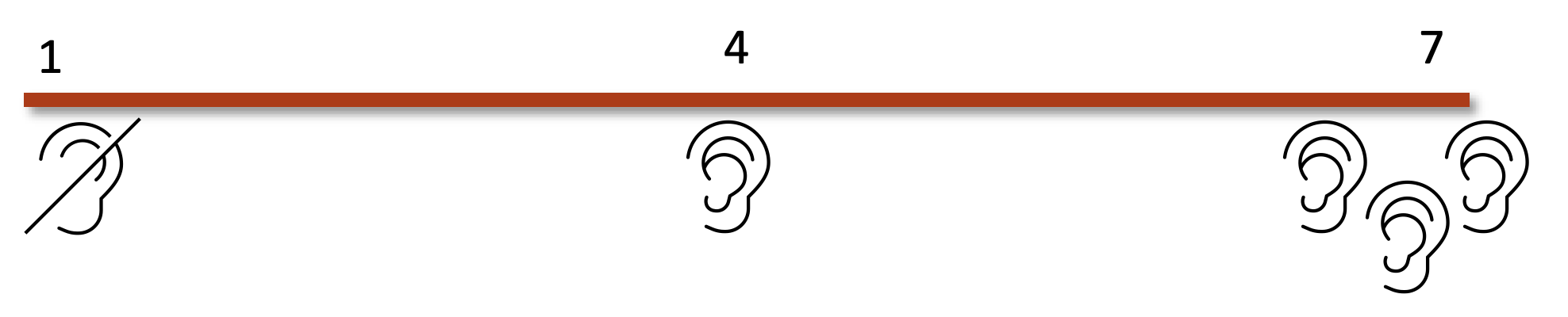


- On a scale of 1 to 7, 1 being “I felt overwhelmed by the discussions” and 7 “I felt I was making a difference”, *how would you rate your feeling of empowerment during the session*?


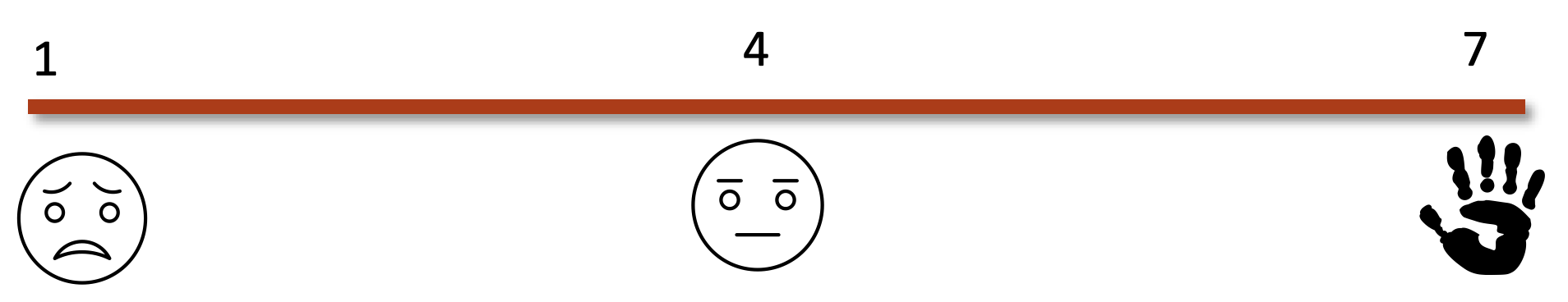


Appendix D – Semi-structured Exit interview questions

- How was your experience in general?
- What would you need to improve your experience?
- What are/were the barriers to your participation?
- Did you wish you had additional skills or knowledge? If any, which ones?

Appendix E – Canvas output of the conversation groups
