# Supplementary material for: Mapping the flow of knowledge as guidance for ethics implementation in medical AI: A qualitative study
Source: PLoS One. 2023 Nov 2;18(11):e0288448. doi: 10.1371/journal.pone.0288448 (PMC10621848; doi:10.1371/journal.pone.0288448)
Supplement: S1 File — Prior study data. Data from a prior study upon which this study is built. (DOCX) [file pone.0288448.s002.docx]

## Methods

A participatory process about a fictitious COVID-19 AI app to​ (1) explore the assumptions, expectations, and perspectives of a range of stakeholders of the GP-Patient-AIHA system in relation to ethics principles and assess the gaps and tension between them, and (2) gain an understanding of the influence of the research process on the participants’ worldviews in relation to ethics principles.​

Three-phase process: (1) Individual interview, (2) focus groups, (3) exit interviews.

# Results

| Gender | 10 males, 8 females |
| --- | --- |
| Age | median = 45 years; span = 23-82 years; SD=17.7 years |
| Location | 10 in urban areas, 6 in semi-rural areas, 2 overseas |
| Professional background represented | GP, nurse, surgeon, technology executive, bio-medical engineer, systems engineer, social worker, retail worker, lecturer, administrator, strategy consultant |
| Cultural background represented | Anglo-Saxon, Chinese, South-Asian, European, and Middle Eastern, with 8 participants speaking another language in addition to English |
| Highest level of education | 1 HS diploma, 5 Bachelors, 6 Masters, 6 PhDs |
| Technology savviness | All except one own a personal computer, all own a smartphone, the self-rated level of knowledge about AI is on average 3.6/7 ranging from 1 (no knowledge) to 7 (AI programmer) with a SD of 1.9, and the self-rated level of affinity with technology is on average 5.4 ranging from 3 to 7 with a SD of 0.97. |

## THEMES - OVERVIEW

Seven thematic 1) assumptions about health and the GP-patient relationship, 2) AIHA enhancing the GP-patient relationship, 3) overreliance on AI, 4) AIHA redistributes decision-making responsibility, 5) consumers’ AIHA knowledge, 6) AIHI data curation responsibility, and 7) consultation about AIHA as education.

## THEME 1: ASSUMPTIONS ABOUT HEALTH AND THE GP-PATIENT RELATIONSHIP

**Table 2** Excerpts about assumptions about health and GP-patient relationship

| Type | Description | Example |
| --- | --- | --- |
| GP-patient relationship and trust | GP allowing for the patient to take responsibility for one’s health | I think it's how [my GP’s] perception of health and wellbeing is really mine. She lets me be the driver of what is important to me, she only works on things that are important to me that I seek her out about. […] she's one of the rare people in this space that allows me to drive on my health and wellbeing issue. (Patient/P10). |
| GP-patient relationship and trust | Earning trust through questioning and checking information | I go to see a GP and I asked the GP, so what is this? And she says, Well, this is this, and then I will go on, I say what is this? And I'll check out the data? And I asked my GP? You sure? Why do you think it is this? So there's always this conversation going on in order to earn my trust? (Patient/P1) |
| GP-patient relationship and responsibility | A GP takes responsibility for the poor experience of a patient at the surgery. | I've had if it's someone I trust, it's good. But I guess I tend to I've had a bad experience. And it's made me realize that don't necessarily trust the GP and go for a second opinion, if you need. The, unfortunately, my last interaction with the GP was probably not good, because I was waiting for about an hour or something. And I was quite cranky, by the time I went in. And I was determined not to give the She said, Oh, I'm sorry. You know, I'm really sorry to keep you waiting and I was determined not to give the smile. That's all right. So I didn't give that ‘it is alright smile’. And she picked up on that and said, you know, really, I'm sorry, just you know, sometimes it just happens like this. And that made me even angrier. And so I said, Look, you know, it's just not being told, I could have been doing lots of things in that 50 minutes. So it's the lack of information and control of my own life. And anyway, she had had a really hard day and was nearly in tears. And then I was nearly in tears. And she well and she said, Look, I'm really sorry, is there anything I can do? And that blew me away? You know, you know, can I talk to the receptionist and well Yeah, I assume try and solve this problem. And, and I said, Oh, that's the first time any doctors ever offered to solve a problem? She said, Oh, that's terrible. That's probably when we were both nearly crying. I said that at the end, she did the consult. And yeah. Yeah. And then at the end, I wanted to make the point that, you know, thank you very much for actually taking this seriously. And it's the first time anyone's ever actually wanted to address the problem. And yeah, and then I've just kind of feel bad about it. And I haven't gone back. (Patient/P4) |
| Health indicators | What health is for participants is multi-dimensional and relational to their environment and themselves | - dimensions of well-being, and mental health such as “tranquillity” (Tech/P13), - the relationship one has with one’s disease or condition (Patient/P6, P18) - a sense of harmony with oneself and one’s surrounding (Patient/P18), - a sense of being part of a community (GP/P3, Patient/P2).   that sense of well-being, it's not measuring any particular part of you or anything like that. It's rather a whole concept (Patient/P18).  the resource to live meaningfully and in a control, like having a positive control over your own life (GP/P3) |

##

## THEME 2: AIHA enhancing the GP-PATIENT RELATIONSHIP

**Table 3** Interviews excerpts about GP-patient relationship enhancements

| Type | Description | Example |
| --- | --- | --- |
| GP-patient relationship dissatisfaction | The nature of the relationship was perceived by patients as changing, and being less personal and more commercial than desired | Well, the quality of relationship or the type of relationship with GP has changed quite dramatically. Over the last 20 years, 20 years ago, I would describe the GP as one of my best friends and, and colleagues. And when we met on the street, we will immediately be able to chat about how my health was or how his health was. But nowadays, it's a much more formal relationship, almost the point where you avert your eyes, if you pass in the street, there is no particular bond. The doctor client relationship patient relationship has come more of a service relationship. […] I think it's been the evolution of, of the medical clinic, which has become more business orientated, than well-being orientated. So for example, a sign in my latest GP office says, If you have more than one question for the doctor, make a second appointment. (Patient/P18) |
| GP-patient relationship desired characteristics | Long-term relationship with patient is important for a holistic approach | The relation for me with between the GP and a patient has to be personal, [it] has to be that the GP knows you follows you over time. (GP-tech/P16) |
| GP-patient relationship challenges | GP mobility can impede the development of a long-term GP-patient relationship | Doctors work across different practices or just being hop on and off depending on the place. So even if you spend a lot of time with the patient, in a single encounter, if you are not able to follow for, for a certain amount of time this this relationship is really hard to develop much farther. (GP-tech/P16) |
| GP-patient relationship challenges | GP turned into clerks because of technology | That's what we've done with technology, we turn doctors into clerical workers for at least half of their day and taken away the time to actually talk with their patients about health. (GP-Tech/P11) |
| GP-patient relationship and technology expectations | AI would alleviate the clerical burden | … if we can automate all of those [keyboard clicks], […] then I could spend more time with the patient. What I won’t like is that my interaction with the patient in the end, it’s an interaction with the output from the machine. I still want to see the patient and having this output […] then spending less time creating the background myself and having more time with a patient. (GP-tech/P16) |
| GP-patient relationship and technology expectations | AI could lower access barriers to care | I think a lot of people, well, some people, particularly in low socio-economic areas, will put off going to the doctor for medical issues, because they're busy, or they can't afford it, or they just are uncomfortable about going to the doctor. So this might reduce the barriers, if people could interact, easily and quickly, initially with an artificial system. (Patient/P17) |
| GP-patient relationship and recovery | GP-patient relationship is part of the recovery from ailments. | I think a lot of our care is, is social and encouraging and picking up little things like I think it's time we move into a nursing home. Well, I think it's time we've got extra help for you that those incidental bits of care. And a lot of elderly patients are quite thoughtful. And I know they come for a chat, but they will also try not to take our time. Even though we say come and see us. (GP/P3) |
| GP-patient relationship and technology | AI to provide space for GP to focus on empathetic care by relieving the clerical burden. | I think a lot of stuff that GPs […] are just very routine. And a lot […] removes the empathy element. Yeah. So, it in a lot of the information could be replaced by AI. Because, but, but what they need to embrace is the more empathy to use that information to provide that more empathy, empathetic care. (Tech/P15) |

## THEME 3: OVERRELIANCE ON AI

**Table 4** Interviews excerpts about AI overreliance

| Type | Description | Example |
| --- | --- | --- |
| Patient overreliance on AI | AI device user can become obsessed with the output of the device. | You can become a bit obsessed. So whether it's COVID […] just watching my phone, what's my, what's my pulse doing now? I've got to get it, you know, you can become over vigilant. Yeah, yeah, bit neurotic it can, you know, maybe you get information that just does your head in. And if you just did… a bit like being obsessed about what you eat, and wanting to lose weight, or the more you worry about it. The more your body's gonna store this, I'm gonna store this. (Patient/P4)  I know a lot of people like to monitor these things, daily thing for themselves. But I can also see the misuse, potential misuse of them. So they become obsessive about it and obsessive about instead of you know, that it's that kind of fine line of being present, and just living your life. And you know, becoming scared because your heart rate’s going up. And not knowing why. So it's, you know, I think in this day and age, as we are becoming more and more technology bound, the urge to resist, technology becomes harder. (Patient/P10) |
| GP overreliance on AI | AI accentuates reliance on technology | We become over reliant on the system monitoring without the human, if the human is just waiting for the system to say, there's an issue here. (GP/P2) |
| GP overreliance on AI | GP could be losing competency and possibly not needing same depth of training | [AI diagnosis example] kind of rise to me concern that doctors might in a sense, lose competence, competency because they’re perhaps more and more simply reading the diagnosis from an AI process. That they’ve developed kind of supreme confidence in AI […] They won’t even need to be trained apart from basics or something. (Patient/P4) |
| Health agencies overreliance on AI | Overreliance on data from AIHA could lead to a loss of context of the data. | There's also a risk that, you know, there's an over reliance on this sort of data without actually talking to people about what's going on. So that relationship, I mean, I don't know how great that relationship is now. But it could become even more distanced by using the system as like an intermediary, which is still only capturing part of the picture. (Patient/P7) |

##

## THEME 4: AIHA Redistributes DECISION-MAKING rESPONSIBility

**Table 5** Interviews excerpts about responsibility shifting.

| Type | Description | Example |
| --- | --- | --- |
| Patient responsibility | Patient giving away one’s responsibility for one’s health to the AI | I only worry about other people who use it as a form of escape. ‘I don't have to take responsibility for my health, the machine will tell me what to do’. (Patient/P1). |
| GP responsibility | GP abdicating one’s responsibility to AI | It's becoming an external authority, which allows the doctor to abdicate particular responsibility for things because you always say, well, that's what the AI product indicated. (Patient/P18) |

##

## THEME 5: CONSUMERs’ AIHA knowledge

**Table 6** Excerpts about AI app consumers enablement

| Type | Description | Example |
| --- | --- | --- |
| GP and AI conflict | When GP disagrees with AI diagnosis, GP to continue investigating | I don't see a problem with that. As a doctor, you would, you often have situations where you've got a piece of clinical information, this is what I've got wrong. And you do an examination, or you find something else. And then you go and do the tests that are needed. Because your brain is telling you different things about so this is the machine telling you one thing and you think it's something else, so you've got to do some extra things on it. (GP/P12)  If you have another input of information that does not confirm your beliefs, then it’s going to be tricky to manage. (GP-Tech/P16). |
| GP AI education | GP needs to understand AI to not be blind followers | With an AI tool, we really have to understand exactly what it’s telling us to interpret it and not just be blind followers. (GP/P3) |
| GP AI education | AI knowing GP versus AI naïve GP could create socio-economic disparities. | It could be that there's just a bunch of GPs that are like, we don't do AI here. So if you want AI related things, like [..] we don't have any house nurse, so we don't do blood tests here, […] I suppose that there's potentially like, this whole other layer of accessibility […]. Does [having a GP that has that AI knowledge] come with like, a socio-economic layer of where you live? Or where that GP is? (Patient/P7) |
| AI as a GP aid | AI helping GP keep up to date with latest findings | The potential for a doctor, to be able to keep up with every advance in every situation is not possible. Having a machine that could, that would have all the information in it, including only up to date information about new evolving diseases would mean that my lack of ability despite all my best attempts to keep up to date, my lack of ability to keep up to date with absolutely everything would be greatly facilitated by that machine. (GP/P12) |
| AI as GP aid to draw from colleagues | A GP augmented with AI is like a GP tapping into the collective knowledge of many GPs | Because the AI can draw collective intelligence and experience across everybody that's been working within that field over a period of time, […] using a training data set that has the outcome decision that's been made across a whole bunch of different conditions. So you kind of ironing out any error bias or whatever, balancing those out. So that you kind of take those away and you get the collective kind of intelligence of multiple physicians when you're building the kind of the AI algorithm. So I think as an individual that's kind of potentially quite beneficial to know that […] it's not just the clinical experience of the individual physician that you're dealing with but they are able to lean on what's been developed through the AI, from the intelligence across the whole field over a period of time. (Patient/P5) |
| Patient education about AI | Communications about AI output to patients could draw inspiration from the way information is conveyed for medical drugs. | Is this not what we face most of the time, except, you know, when given the drug we should be given the information that there's a one in million chance of a blood clot and blah million in of have a something else or a stroke with it or something. I'm not sure that as consumers, we really take that information onboard […] that's all come from complex statistical analysis. You know, you know, complex stuff, does it matter whether it was an AI algorithm analysis of your colonoscopy, or whether it's from years of data, or whatever the background research was on a particular drug, where in the end, I have that choice, I don't think it would matter to me if I particularly if I understood where the data was coming from. (Patient/P4) |
| Patient education about AI and autonomy | Important to understand how to make an informed decision to preserve control over’s one health. | I wouldn't care, I'd like to have the information though. I like to have information. And then I can make an informed choice. Because I, as I've got older, I've got a very strong health locus of control. I like to know what's going on. And then I can make an informed choice. But I don't like the unknowns. I do like to, to sort of have the detail. (Patient/P6)  This is a tool. There will be doctors involved, there will be oversight, just to try and make it as transparent as possible. You can't explain to people this is exactly how it works. It's too complicated. But you can say these are the sorts of principles it's based on: lots of information, looking at developing patterns, and those patterns […] being used to help make decisions. And I think people can generally understand that. (Patient/P17) |
| AI interpreter for clinician and patient | Possible need for a new function of AI results interpreter | Could you imagine in your clinic that you would employ someone with who is an AI engineer or an expert in that area because the doctors would need to have a reference person if you like that they are controlling and feel like it's part of the team to ensure you know the appropriate advice is …(Patient/P18)  Yes. Yeah, I feel like you need a whole new person or project. Yeah, it's, yeah, we're not educated in this field. (GP/P3) |

##

## THEME 6: AIHA data CURATION responsibility

### Data quality

**Table 7** Excerpts about data quality

| Type | Description | Example |
| --- | --- | --- |
| GP data input | GP inputting data is better for data quality | I think the doctors should be the one that inputs the data in the system, not the patient, because maybe sometimes patients may have different understanding of a health issue as well. So maybe they have like some symptoms that two different people evaluate as different levels, but the doctor may be able to have a better understanding of their symptoms and have a more like, equal assessment of both of them. (Tech/P8)  Now, I know that machine learning, is supposed to enable us to become more and more finesse about an individual person. But that requires a lot of time to feed individual data in for every person, and if they need me to make another 15-minute appointment for my second issue. They certainly haven't got time to put my uniqueness into an AI machine. (Patient/P18) |
| Patient data input | Patient inputting data may exaggerate symptoms for faster access to care | People who want to, who play with the system to get what they want, not what they really deserve (Tech/P8) |
| AI quality matching GP expectations | Data quality needs to match the assumptions or expectations of the GP or it could lead to misinterpretation | There's two points at which the quality of the data really comes up. One is when you're actually doing the engineering to create the models. […] I think the second place where it shows up is, is if you put a model into production, that is to say it has now become a decision aid somewhere in some healthcare system, it's helping a GP, who doesn't know anything about AI, doesn't really think much about data. […] does the data quality on which that this recommendation is going to be made match the data quality assumptions on which the model is built? […] every model needs to be allowed to say, I can't give you an answer. Or the only answer I can give you has error bars so large that you're going to disregard it anyway. (GP-tech/P11) |

###

### Data usage

**Table 8** Excerpts about data usage

| Type | Description | Example |
| --- | --- | --- |
| Usage of data | Consider how data would be used in the broader context of the healthcare system | So, what are we going to do with it? Just turn it loose on the world. So that that, you know, just those people who happen to have the right kind of watch now get early warning of COVID? Or are we going to turn it in to you know, something that is systematically useful? across the healthcare system? If you don't have an answer to that question, you have no business building the thing. (GP-Tech/P11). |
| Repurpose of data for commercial usage | Data could be reused by commercial partners to promote products | I can see that my health data will go to some pharmaceutical company who will want to push me its brand of whatever right whatever I may be whatever problems we are having. And what I get will be whichever advertise. So I will get on the side. I get a lot of advertisement try this. Try that try this. Try that. that I think will be very bad. […] It's very hard for data not to be commercialize. People do this for a profit […] every time there is an opportunity for making more money, somebody will come in. That's been the world so far, and I can't see it being any other ways. (Patient/P1) |
| Data used by government | Data could be used by government to determine access to selected services. | Well, I mean, I think in an ideal world, it's not a data scavenging business model. But then what are your other options? Like a some sort of subscription based model where like, the patient is actually paying money to have this app? will probably be less lucrative? Well, depends. I don't know, who knows. So the patient's actually paying for the service directly, then that is another option, I suppose. If it was government funded, that's another option. But then, you know, that's also connected in with governments owning data and connecting that data to your welfare payments or something[…]Well, I think again, it comes back to like the link ability of the data set, because if they start linking that data set with your welfare payments or with your Medicare rebates or with I don't know, some other access to some government scheme, you know, like everyone's getting Dine and wine or whatever vouchers or something but you're you've had COVID so they don't want you dining or wining. (Patient/P7) |
| Data used for profiling | Data used for profiling losing sight of context is concern. | [Data] provided could be misused by turning them into profiling someone. Because they might find an interesting case, you know, an interesting profile, and that, that shown as a, and that person is still having COVID, for example. So, it's just like this AstraZeneca thing. where, you know, you got blood clot, I mean, it's now labelled as everybody above 50 can get it […] I think it could well be mis-concluded. (Tech/P15) |
| Data serving political agendas | Data used to serve the agenda of some political entity is a concern. | It depends whether the public health agencies are political agency or not, if they run as independent health agencies by health professionals, I don't have too much worry about it. The only concern I have is that their KPIs may demand certain outcomes, and therefore they read the data to suit these personal agendas and assumptions and so on. (Patient/P18) |
| Data as GP activity monitoring | Data used to monitor GPs engagement with the technology is a concern. | a scrutiny of the doctor for the advice or lack of advice or engagement that they have with the information that's coming. So from their point of view, it could be seen as Big Brother or big sister looking over the shoulder. For the patient, it could easily become that they get stereotyped because of the information that's had which is such a limited amount of, of that whole person, but it could be used in to put them into a stereotype cluster and dealt with accordingly. (Patient/P18). |

###

### Sharing data as an act of solidarity

**Table 9** Excerpts about solidarity

| Type | Description | Example |
| --- | --- | --- |
| Sharing data as a long-term investment | Data-sharing seen as a long-term investment | I would trust the system with giving my data to them to develop to, to develop the machine learning system. And that comes from the optimistic side of my brain. So, because I, I believe if we want to have a system that can be used for everyone, they need our data. And if we, like, if we are optimistic that they don't use it for other, like, for advertising or stuff like that I think I'm okay with giving my data to them, but I would not trust with the feedback that the machine learning system gives me for at least a couple of years, or at some point that I, I am assured that the system works properly. (Tech/P8) |
| Sharing data out of solidarity | Sharing data to build a system that would benefit underprivileged people | Going by the current knowledge about COVID-19. You’d have to say that it’d be more like a shotgun blast if you go by the literature, rather than a rifle shot because the people we just don’t know there are so many unknown unknowns that have Having something like this might actually be a research tool rather than a, a diagnostic tool. […] as a research tool then it was good to help someone like yourself trying to understand more effectively in different populations, how COVID manifests itself, then I would be happy to take that on and to have the data when I’m tested positive, forwarded to the people who are running the machine. (Patient/P18) |

###

### Data custodians

**Table 10** Excerpts about data custodians

| Type | Description | Example |
| --- | --- | --- |
| Data brokerage | Data brokerage service to ensure data privacy | Like in the same way you might have a I don’t know, if you’re super wealthy might have some wealth manager, could you have just like a health data manager, someone you trust that understands all the ins and outs of what’s going on? And if need be, can broker you out of the system? Or move you on to a different platform that you know, better aligns with your values or whatever? (Patient/P7) |
| Regulations | Need for continuous monitoring | If we don’t have a continuous monitoring on those systems, I don’t think it will be possible to build that trust” (Tech/P8). |

## THEME 7: consultation about AIHA as EDUCATION

- Most participants also reported learning from their fellow participants.
- Tech/P13 reported how he had not realised how vulnerable patients were in their relationship to a clinician, or an AIHA.
- Patient/P17 related how the experience boosted his confidence that AI can be designed and implemented ethically.
- From the researcher’s point of view, the research raised awareness of the imbalance of knowledge between AI experts and AI consumers.
